# Multivessel Spontaneous Coronary Artery Dissection and Myocarditis: A Rare Case Highlighting the Value of Multimodality Cardiac Imaging

**DOI:** 10.7759/cureus.91407

**Published:** 2025-09-01

**Authors:** Jared C Daddario, Matthew A Tunzi, John-Henry L Dean, Rosco S Gore

**Affiliations:** 1 Internal Medicine, San Antonio Uniformed Services Health Education Consortium, Fort Sam Houston, USA; 2 Cardiology, San Antonio Uniformed Services Health Education Consortium, Fort Sam Houston, USA

**Keywords:** cardiac magnetic resonance imaging (mri), coronary computed tomography angiography (ccta), intravascular ultrasound (ivus), invasive coronary angiography, late gadolinium enhancement, myocarditis, nonatherosclerotic, spontaneous coronary artery dissection (scad)

## Abstract

Spontaneous coronary artery dissection (SCAD), once considered too rare to study, is now recognized as an important nonatherosclerotic and nontraumatic cause of myocardial infarction, especially in young women. However, it is often challenging to differentiate from alternative etiologies of chest discomfort, such as atherosclerotic acute coronary syndromes or myocarditis. Here, we describe the unique case of a young woman who presented with an acute chest pain syndrome and was found to have co-occurring myocarditis and multivessel SCAD. The use of multimodality imaging studies to include transthoracic echocardiogram (TTE), cardiac magnetic resonance imaging (MRI), coronary computed tomography angiography (CCTA), invasive coronary angiography (ICA), and intravascular ultrasound (IVUS) delivered the confirmation of concomitant diagnoses which were consistent with the clinical presentation. This case highlights the critical role of multimodality imaging in the diagnosis and management of coexistent cardiac pathologies.

## Introduction

Acute myocarditis, by definition, is inflammation of the myocardium and generally consists of infectious (viral, bacterial, fungal, protozoal) and non-infectious etiologies. Non-infectious causes may include autoimmune, neoplastic, trauma, and drug-induced. Myocarditis, and specifically myopericarditis, incidence is unclear given the multitude of etiologies. It may be subclinical with mild or absent symptoms. Other cases may show a clear cardiac etiology or be masked by a large inflammatory response with systemic manifestations from the underlying cause. One example may include a severe viral upper respiratory infection [1].

In contrast, spontaneous coronary artery dissection (SCAD) is a nonatherosclerotic cause of acute coronary syndrome (ACS) that primarily affects young to middle-aged women, often without traditional cardiovascular risk factors, and is characterized by a separation of layers of the epicardial coronary arteries by intramural hematoma or intimal tear [2]. Despite improving recognition of SCAD in the clinical setting, its acute presentation can mimic other conditions, such as myocarditis, which can complicate diagnosis and management [1,3,4]. The diagnostic process becomes even more complex when there is preexisting coronary artery disease (CAD) and a history of prior percutaneous coronary intervention (PCI).

While the pathophysiology of both cardiac conditions of SCAD and myocarditis differs, the manifestation is classically that of a chest pain syndrome. This case highlights the diagnostic challenges that arise when overlapping clinical features obscure the underlying pathology, ultimately necessitating multimodality imaging to refine diagnostic accuracy and reveal the complex etiology of the patient's presentation.

## Case presentation

A 37-year-old woman with a history of non-ST-segment elevation myocardial infarction (NSTEMI) status post PCI to the distal right coronary artery (RCA), hyperlipidemia, and a family history of premature CAD was admitted to the inpatient cardiology service for the evaluation of acute-onset chest discomfort. She had a notable cardiac family history of myocardial infarction (MI) status post two-vessel coronary artery bypass graft (CABG) in her father. On the day of presentation, she awoke from sleep with acute-onset chest pain, which she characterized as a burning sensation with chest pressure that was worse with lying flat. Additionally, she described symptoms of lightheadedness and dizziness associated with these episodes. She denied recent illness, sick contacts, or travel outside the local area.

In the emergency department, serial electrocardiograms (ECGs) demonstrated normal sinus rhythm without evidence of ischemia. Limited bedside transthoracic echocardiogram (TTE) showed no regional wall motion abnormalities. High-sensitivity troponin T peaked at 120 ng/L and subsequently trended down. Inflammatory markers that include erythrocyte sedimentation rate (ESR) and C-reactive protein (CRP) were negative.

**Figure 1:**
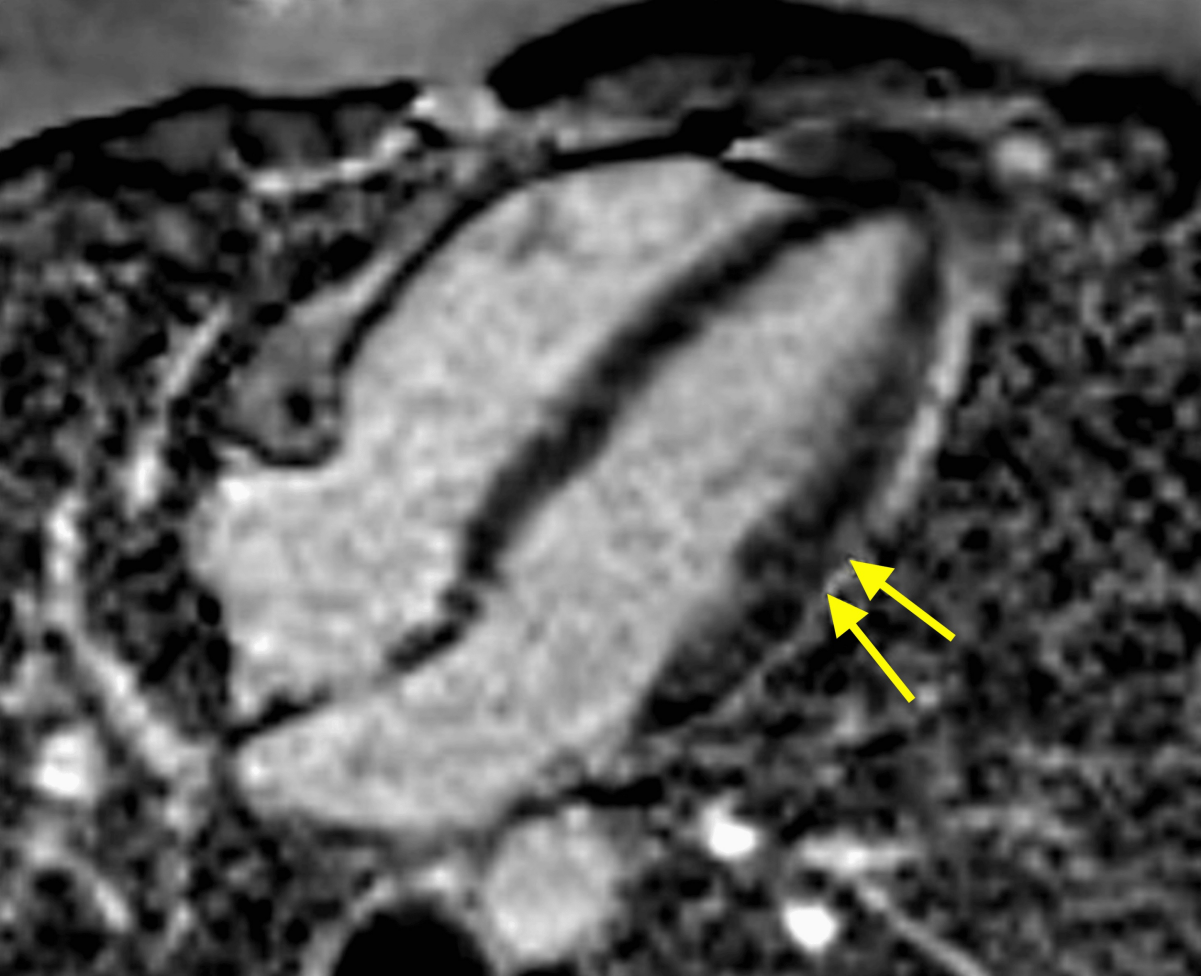
Four-chamber view demonstrating focal mid-myocardial late gadolinium enhancement in the mid-lateral left ventricle consistent with nonischemic myocardial injury and subtle focal myocarditis Yellow arrows: Late gadolinium enhancement of the patient's mid-lateral left ventricle

**Figure 2:**
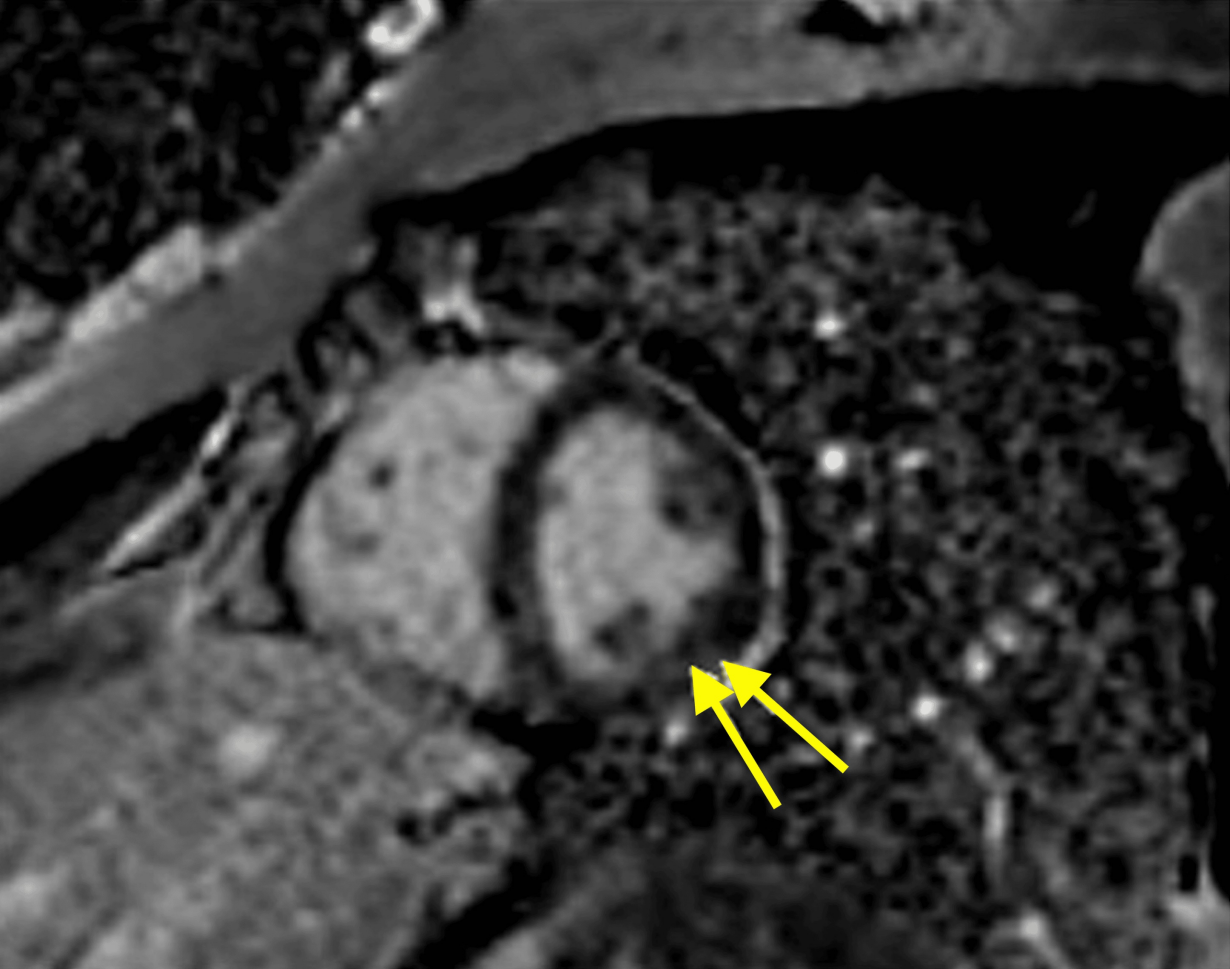
Short-axis view demonstrating focal mid-myocardial late gadolinium enhancement in the mid-lateral left ventricle consistent with nonischemic myocardial injury and subtle focal myocarditis Yellow arrows: Late gadolinium enhancement of the patient's mid-lateral left ventricle

Initial presentation was concerning for myocarditis, with lower clinical suspicion for ACS given her nonanginal chest pain and the lack of ischemic changes on ECG and bedside echocardiogram. No clinical features of coronary vasospasm or microvascular disease were present. She was admitted to the inpatient cardiology service with continuous telemetry and planned further workup with cardiac magnetic resonance imaging (MRI). Cardiac MRI completed on the night following admission identified focal mid-myocardial late gadolinium enhancement in the mid-lateral left ventricle and focal myocardial edema by T2-weighted images in the same area, with otherwise normal negative T1 and T2 map times (Figures 1-2). Findings were consistent with subtle focal myocarditis. TTE on day 1 demonstrated normal left ventricular systolic function with an ejection fraction of 55-60% and trivial pericardial effusion.

**Figure 3:**
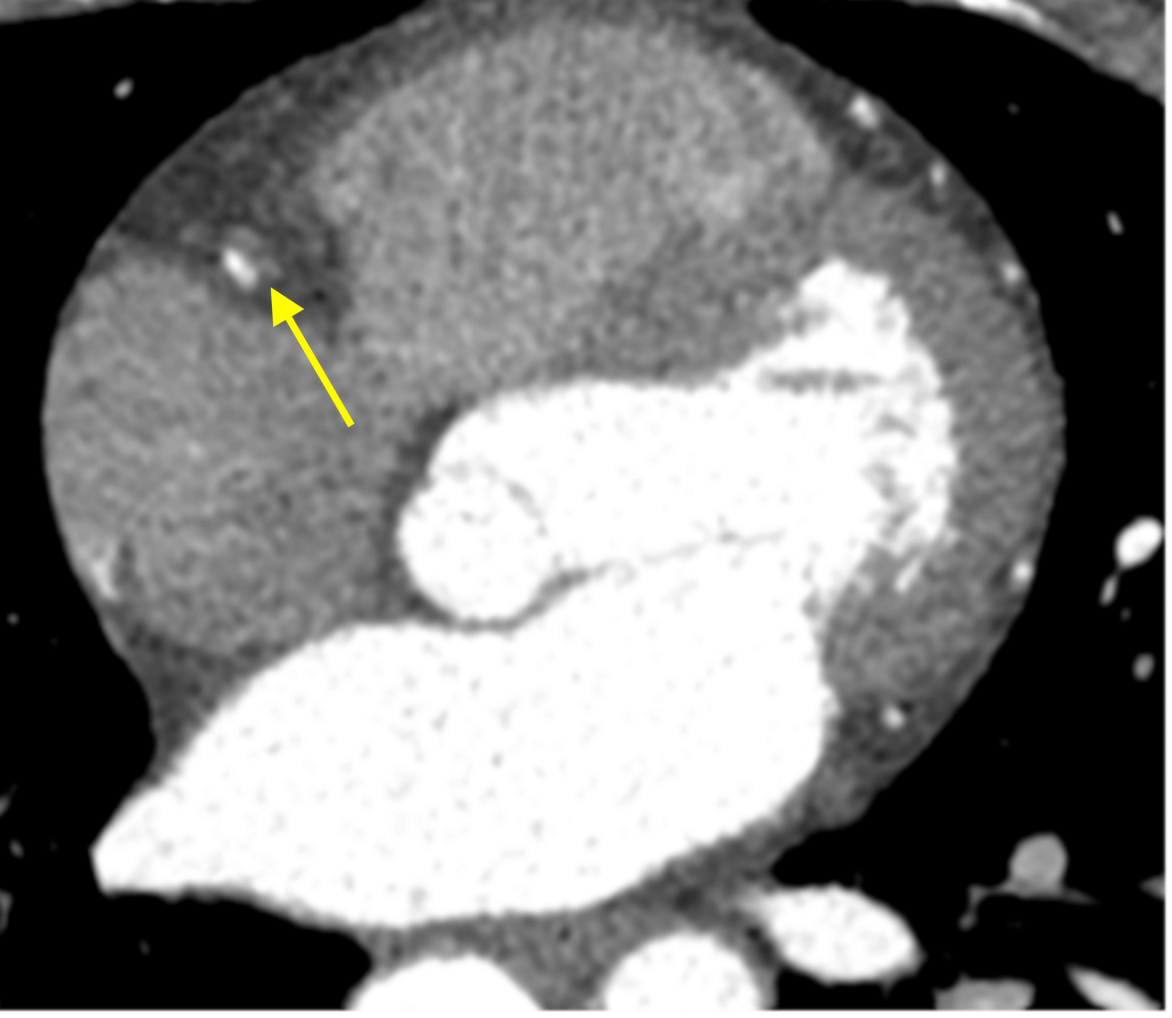
Axial view of SCAD (yellow arrow) in the RCA on CCTA SCAD: spontaneous coronary artery dissection; RCA: right coronary artery; CCTA: coronary computed tomography angiography

**Figure 4:**
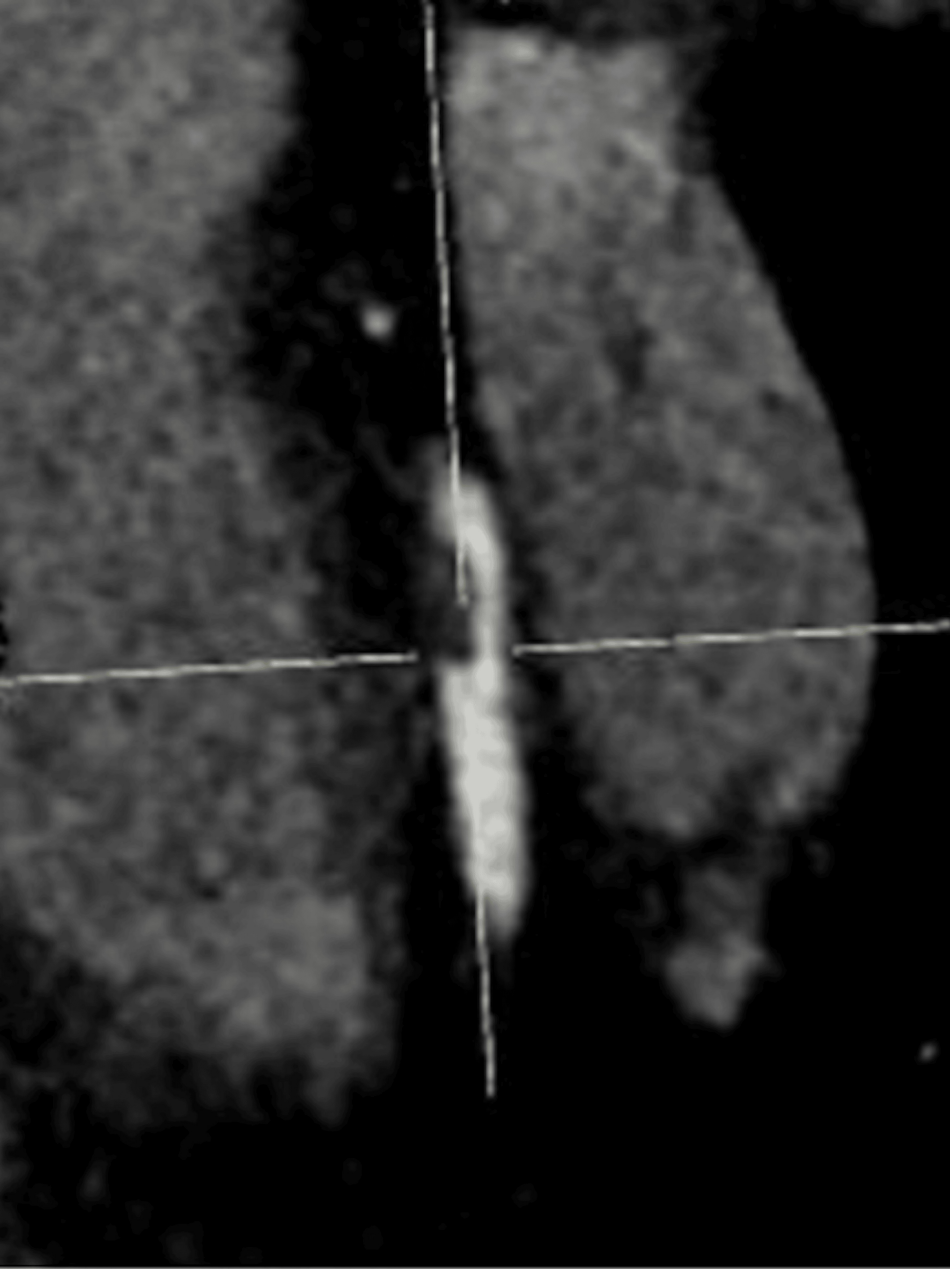
Double oblique view demonstrating the non-invasive nature of CCTA to visualize the proximal RCA dissection and hematoma RCA: right coronary artery; CCTA: coronary computed tomography angiography

Despite the evidence for myocarditis, non-invasive coronary angiography via a coronary computed tomography angiography (CCTA) was pursued to evaluate coronary pathology, given her known history of CAD and in accordance with the 2015 European Society of Cardiology (ESC) Pericarditis Guidelines [1]. Invasive coronary angiography (ICA) was not pursued as there was low clinical suspicion for ACS and her symptoms had resolved. Ultimately, the CCTA demonstrated severe stenosis (70-99%) of the proximal RCA with negative remodeling and characteristics suggestive of an intramural hematoma with thrombus secondary to coronary artery dissection (Figures 3-4).

**Figure 5:**
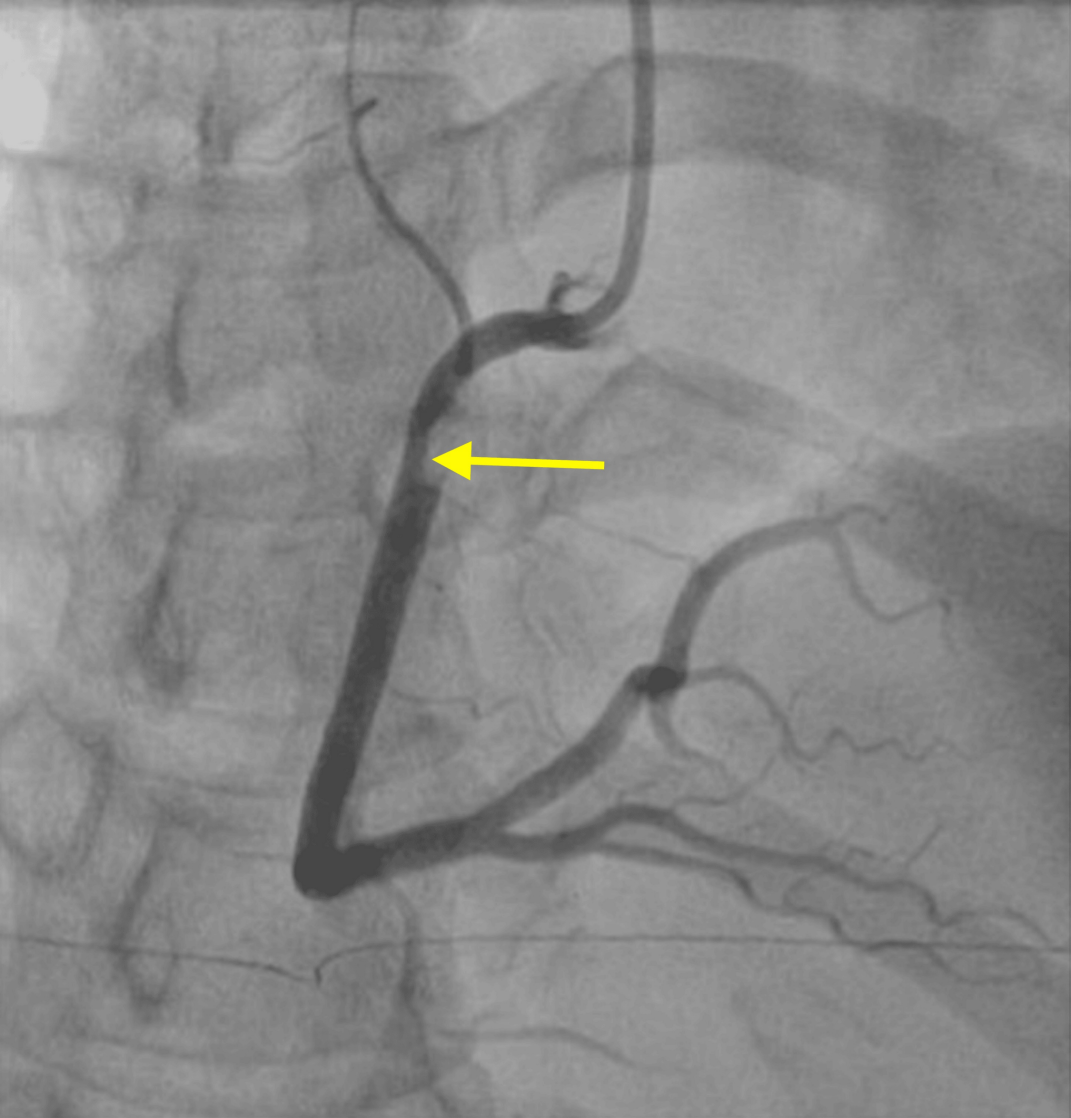
SCAD (yellow arrow) of the RCA on ICA SCAD: spontaneous coronary artery dissection; RCA: right coronary artery; ICA: invasive coronary angiography

**Figure 6:**
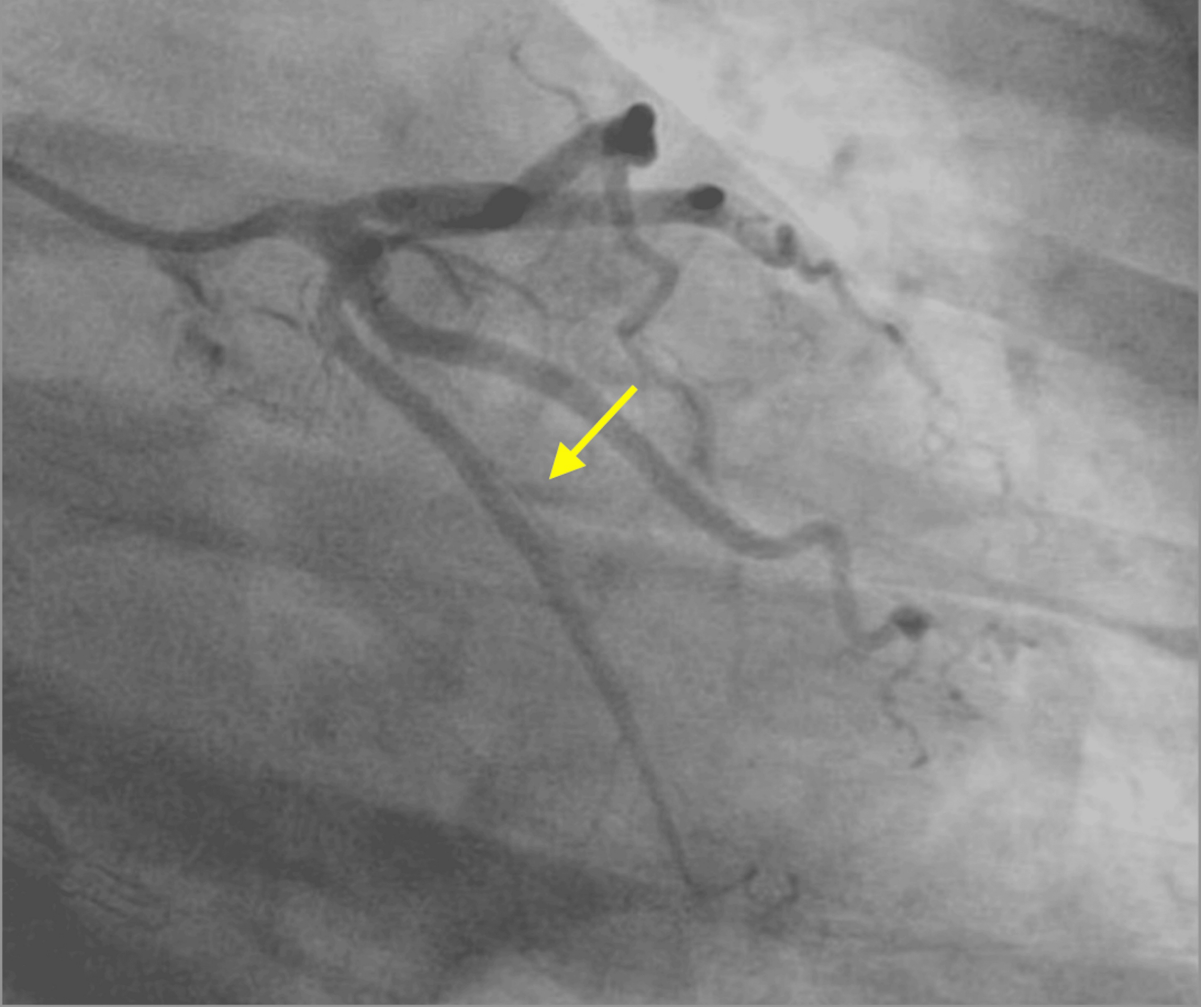
SCAD (yellow arrow) of the OM2 on ICA SCAD: spontaneous coronary artery dissection; OM2: second obtuse marginal artery; ICA: invasive coronary angiography

While initially treated with colchicine and high-dose aspirin given the aforementioned positional nature of chest discomfort, this was later discontinued after identifying SCAD, and she was started on medical therapy with aspirin 81 mg daily in addition to high-intensity statin and low-dose colchicine given her history of CAD. The following day, CCTA findings ultimately prompted ICA which further supported the diagnosis of multivessel SCAD within the proximal RCA (Figure 5) and the second obtuse marginal artery dissection (Figure 6). Intravascular ultrasound (IVUS) was utilized to confirm dissection in the RCA.

No acute percutaneous interventions were pursued, given her overall stable condition. Comprehensive non-invasive angiography was completed prior to hospital discharge, with no evidence of fibromuscular dysplasia in the assessed vascular beds. Studies included magnetic resonance angiography (MRA) of the chest, abdomen, and pelvis and computed tomography angiography of the neck and brain. Further, pregnancy testing was negative, and she had no history concerning for connective tissue disorders, autoimmune disorders, or active hormone use. Of note, she received fertility treatment six months prior, which can be a risk factor given the hormonal effects on the integrity of the vasculature [2]. She was counseled on activity restrictions and pregnancy counseling with a plan for repeat surveillance imaging and follow-up in the outpatient setting. At discharge, she was symptom-free and required no antianginal therapy. She was prescribed guideline-directed management and medical therapy for her CAD, and she was advised to complete 3-6 months of exercise restriction for recovery from myocarditis and SCAD. Following the completion of the exercise restriction, it was recommended that she begin cardiac rehabilitation.

**Figure 7:**
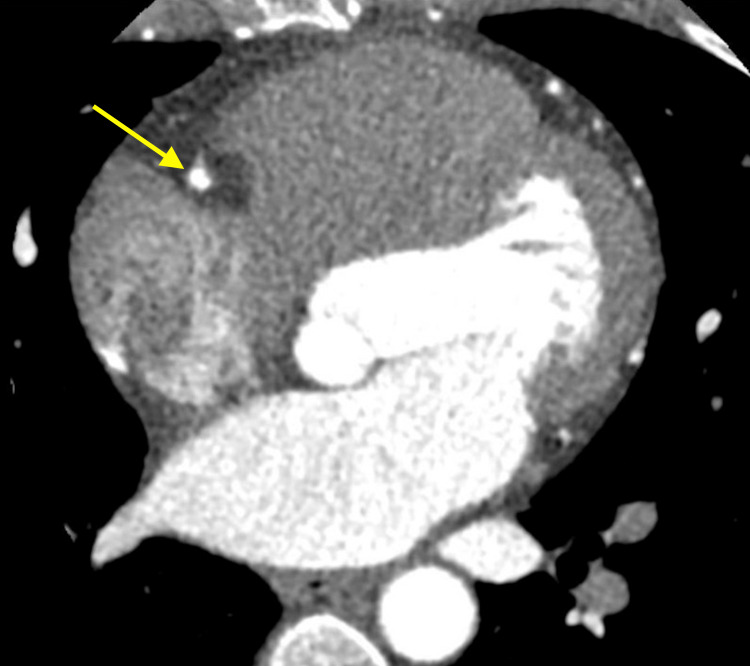
Repeat CCTA with mild RCA stenosis (yellow arrow), highlighting the spontaneous resolution of SCAD and prior severe RCA stenosis CCTA: coronary computed tomography angiography; RCA: right coronary artery; SCAD: spontaneous coronary artery dissection

Outpatient surveillance CCTA was completed roughly 60 days after discharge and demonstrated improvement in the SCAD findings. The previously severe (70-99%) proximal RCA stenosis improved to mild stenosis (1-24%) (Figure 7) on repeat CCTA imaging. Repeat cardiac MRI was also recommended after 3-6 months to assess for resolution but was ultimately not performed as the patient was lost to follow-up.

## Discussion

Although late gadolinium enhancement may be present in both myocarditis and SCAD, cardiac MRI can often delineate the myocardial inflammation and edema based on the degree of inflammation and layers affected. Late gadolinium enhancement in myocarditis is classically located in the sub-epicardium or mid-myocardium, while delayed enhancement in SCAD is characteristically subendocardial or transmural and localized to a coronary distribution [2,3]. Treatment for cases of mild myocarditis and low-risk SCAD is similar, both founded on supportive care, early avoidance of high-intensity exercise, and optimal medical therapy [1]. While there was radiographic evidence of focal subtle myocarditis, her known CAD prompted further evaluation and subsequently revealed multivessel SCAD.

With SCAD, the two hypotheses most discussed include the "inside-out" and "outside-in" mechanisms. The "inside-out" hypothesis states that an intimal tear and creation of a dissection flap create conditions for coronary blood to fill and propagate a false lumen. The second, "outside-in" hypothesis, theorizes that hemorrhage into the tunica media causes compression of the vessel with intramural hematoma [4,5]. There are also different classifications of SCAD to include type 1, type 2A, type 2B, type 3, and type 4. Type 1 is commonly described as an arterial dissection with multiple radiolucent lumens visible on ICA as a result of an intimal tear. Type 2 is the most common form of SCAD and can be categorized into types 2A and 2B. In type 2, there is diffuse narrowing of the artery due to an intramural hematoma. No intimal tear is observed. Type 2A generally reflects a long lesion, with abrupt caliber change and a return to normal caliber. Type 2B, on the other hand, has narrowing which persists to the end of the artery. In type 3, there is also an intramural hematoma; however, it is usually short in length, giving the appearance of a ruptured atherosclerotic plaque. This is the least common. Lastly, in type 4, there is complete occlusion of the vessel similar to that of a thromboembolic occlusion [2].

SCAD is a driving cause of MI in young persons with a predominance in women. In fact, SCAD is the cause of ACS in up to 35% of all women less than the age of 50 years old [2]. MI secondary to SCAD varies significantly from traditional atherosclerotic plaque rupture with its own associated risk factors. For example, SCAD can be a manifestation and even the initial manifestation of fibromuscular dysplasia. Various risk factors include emotional or physical stress, stimulant use, hormonal triggers to include oral contraceptive pills (OCPs) and pregnancy, and inflammatory disorders [2-7]. There are reported familial cases; however, these are primarily seen sporadically. Other conditions associated with SCAD include Ehlers-Danlos syndrome, Marfan syndrome, and Loeys-Dietz syndrome. Specific genetic loci may play a role; however, there are no definitively identified regions to date [2,4,5,7].

The clinical presentation of SCAD varies widely, with patients sometimes presenting with NSTEMI, ST-segment elevation myocardial infarction (STEMI), cardiogenic shock, or ventricular arrhythmias [2,4,7]. High-sensitivity troponin and ECG are often abnormal, though this can vary based on time to presentation and may be negative [2,7]. Unfortunately, there is data suggesting that 25% of female patients who present with ACS secondary to SCAD have experienced similar symptoms without presenting to the emergency department for evaluation [2]. With the potential for normal findings on initial workup, it is imperative to maintain a high index of suspicion to arrive at the correct diagnosis [2].

ICA is the primary diagnostic tool and gold standard as it allows direct visualization with the ability to assess for high-risk anatomy. IVUS and optical coherence tomography (OCT) may be used to confirm findings as well [4,5,7]. IVUS was limited in this case but did demonstrate an RCA dissection and hematoma. OCT was not available at this institution.

CCTA has also become increasingly utilized given its non-invasive nature allowing for the visualization of dissections, flaps, stenosis, and hematoma. Notably, there are overall limitations in spatial resolution and in visualizing distal, small-caliber vessels [5]. CCTA is not recommended in high-risk ACS but can be performed in low- and intermediate-risk patients [4].

Management of SCAD varies from that of atherosclerotic ACS. While atherosclerotic MI may warrant PCI or CABG, medical therapy is the cornerstone for the management of SCAD. Only under specific circumstances would SCAD necessitate PCI or surgery, such as left main disease, proximal multivessel dissection, ventricular arrhythmia, or cardiogenic shock [2,4,5,7]. The risks associated with PCI include propagation of hematoma distally and even affecting proximal vessels via retrograde extension [2,4,7]. While mortality is low, there is an increased risk of recurrence in other vessels [2,4-7]. Multivessel SCAD can occur in noncontiguous arteries which further asserts the idea of avoiding intervention as recurrence may be inevitable in another vessel. Fortunately, dissections generally resolve spontaneously with significant interval improvement on repeat imaging with modalities like CCTA reflecting improved vessel caliber with unimpeded flow [4,5,7]. With activity restriction, cardiac rehabilitation, and counseling on risks of pregnancy, patients with SCAD can safely recover [7].

## Conclusions

This case underscores the importance of maintaining a high index of suspicion for different coronary artery pathologies, even in the setting of clinical and radiological evidence for myocarditis. The demonstrated concomitant pathologies ultimately affected the clinical course and management in this case. Additionally, this case highlights the importance of broadening the differential diagnoses to include SCAD in young women presenting with acute chest pain syndromes, even in the context of prior coronary interventions. Further, it demonstrates the pivotal role of multimodality imaging in improving diagnostic accuracy given the overlapping clinical features of SCAD with other causes of chest pain syndromes such as traditional atherosclerotic ACS or myocarditis.

## References

[1] Adler Y, Charron P, Imazio M (2015). 2015 ESC guidelines for the diagnosis and management of pericardial diseases: the Task Force for the Diagnosis and Management of Pericardial Diseases of the European Society of Cardiology (ESC). Eur Heart J.

[2] Kim ES (2020). Spontaneous coronary-artery dissection. N Engl J Med.

[3] Chandrasekhar J, Thakkar J, Starovoytov A, Mayo J, Saw J (2020). Characteristics of spontaneous coronary artery dissection on cardiac magnetic resonance imaging. Cardiovasc Diagn Ther.

[4] Hayes SN, Kim ES, Saw J (2018). Spontaneous coronary artery dissection: current state of the science: a scientific statement from the American Heart Association. Circulation.

[5] Pergola V, Continisio S, Mantovani F (2023). Spontaneous coronary artery dissection: the emerging role of coronary computed tomography. Eur Heart J Cardiovasc Imaging.

[6] Würdinger M, Cammann VL, Ghadri JR, Templin C (2022). Spontaneous coronary artery dissection: a rare event?. Heart Fail Clin.

[7] Hayes SN, Tweet MS, Adlam D, Kim ES, Gulati R, Price JE, Rose CH (2020). Spontaneous coronary artery dissection: JACC state-of-the-art review. J Am Coll Cardiol.

